# Alternative antibiotic feed additives alleviate pneumonia with inhibiting ACE‐2 expression in the respiratory system of piglets

**DOI:** 10.1002/fsn3.2089

**Published:** 2020-12-27

**Authors:** Chen‐Wen Lu, Sheue‐Er Wang, Wan‐Jhen Wu, Li‐Yu Su, Che‐Hsuan Wang, Pei‐Hwa Wang, Chung‐Hsin Wu

**Affiliations:** ^1^ School of Life Science National Taiwan Normal University Taipei Taiwan; ^2^ Department of Animal Science and Technology National Taiwan University Taipei Taiwan

**Keywords:** alternative antibiotic, angiotensin‐converting enzyme 2, feed additive, piglets, pneumonia

## Abstract

Weaning piglets often suffer from pneumonia during growth, so in general, antibiotics are used by owners to make piglets grow smoothly. However, antibiotics may be accompanied by many side effects such as gastrointestinal discomfort and allergies. The aim of this study was to develop an alternative antibiotic herbal veterinary medicine for alleviating pneumonia in weanling piglets. As observed in the pig ranches, many weanling piglets suffer from the pneumonia and also show high expression of angiotensin‐converting enzyme 2 (ACE‐2) in their respiratory and intestinal tracts. ACE inhibitors have been reported that can decrease pneumonia risk through their main mechanism of action. Thus, we also try to find alternative antibiotic feed additives that can reduce expression of ACE‐2 in piglets. We selected the Guizhi Li‐Zhong Tang Extract Granules (GLZ) as a natural product for piglets. Then, we compared the serum immunoglobulin levels of piglets with sham, tilmicosin antibiotic (TAB), and GLZ treatments. Our results showed that piglets with GLZ treatment had significantly a higher expression of immunoglobulin A and immunoglobulin G but a lower expression of immunoglobulin E than those with sham and TAB treatments. In addition, piglets with GLZ treatment showed obviously low pneumonia incidence than those with sham and TAB treatments. Similarly, piglets with GLZ treatment showed significantly lower expressions of ACE‐2 in their tracheal, bronchial, and lung tissues than those with sham and TAB treatments. GLZ seems to be an alternative ACE inhibitor that can decrease pneumonia risk through inhibiting ACE‐2 expression and alleviating allergies in their respiratory systems. Thus, we suggest that GLZ can be an alternative antibiotic feed additive for weaning piglets.

## Graphical abstract

Schematic diagram illustrates that GLZ treatment can protect the respiratory system of weanling piglets from allergies and respiratory symptoms because of reducing ACE‐2 expression in the respiratory systems.

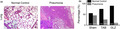

**Highlights**

- Herbal extracts of "Guizhi Li‐Zhong Tang Extract Granules (GLZ)" can be an alternative to antibiotic feed additives for alleviating pneumonia in weanling piglets.
- Piglets with GLZ treatment show better immunity and lower angiotensin‐converting enzyme 2 (ACE‐2) expression in the respiratory system than those with tilmicosin antibiotic and sham treatments.
- GLZ treatment may alleviate respiratory allergies and pneumonia in weanling piglets through inhibiting ACE‐2 expression in the respiratory systems.

## 1 INTRODUCTION

As observed in the pig ranches of Taiwan, some weanling piglets may develop pneumonia with a high expression of angiotensin‐converting enzyme 2 (ACE‐2) in their respiratory and intestinal tracts. ACE‐2 is an entry receptor for the severe acute respiratory syndrome‐associated coronavirus 2 (SARS‐CoV‐2), which caused an outbreak of fatal atypical pneumonia that was eventually called coronavirus disease 19 (COVID‐19; Zhou et al., 2020). High ACE‐2 expression was identified in the epithelial cells of oral mucosa (Xu et al., 2020), upper airway (Jia et al., 2005), and intestinal tract (Lamers et al., 2020), and in lung alveolar cells (Zou et al., 2020). SARS‐CoV‐2 virus infection affects many organ systems in humans but primarily infects the lower respiratory tract, which causes the symptoms of fever, dry cough, and dyspnea (Huang et al., 2020). In addition, headache, dizziness, vomiting, and diarrhea have been observed in patients with COVID‐19 (Shi et al., 2020). Patients with severe COVID‐19 may develop acute respiratory distress syndrome (Wang et al., 2020), which is characterized by difficulty in breathing and low blood oxygen levels that may directly lead to respiratory failure (Li et al., 2020). Some patients with COVID‐19 may die from secondary bacterial and fungal infections because the immune system releases a high level of cytokines and then sepsis results, leading to organ failure (Guan et al., 2020).

Weaning is the most critical period for pig growth, during which piglets must cope with environmental and nutritional stress (Peace et al., 2011). Due to the immature immune system of weaned piglets, these stressors can cause severe oxidative stress and intestinal disorders, thereby leading to poor growth, diarrhea, and other diseases (Heo et al., 2013; Pluske et al., 1997; Yin et al., 2014). Pathogen transmission during pig breeding and growth often damages their respiratory tracts, leading to respiratory diseases. The porcine respiratory disease complex is a pneumonia that causes clinical disease and body weight loss lately in piglets (Chae, 2016). The pathophysiology of pneumonia with a high expression of ACE‐2 in weanling piglets closely resembles that of SARS‐CoV‐2 infection, with aggressive inflammatory responses strongly implicated in the resulting damage to airways. Therefore, disease severity in weanling piglets is due to not only the viral or bacterial infection but also the host response. Symptoms that were noted in piglets before their death were fever, cough and dyspnea, and diarrhea. Among these symptoms, the respiratory disease that involves fatal pneumonia in weanling piglets is the most important health concern for swine producers. Pneumonia is often caused by clinically silent aspiration of oropharyngeal contents. ACE inhibitors can decrease pneumonia risk through their main mechanism of action (Dublin et al., 2012). For avoiding or alleviating pneumonia and diarrhea and improving the growth performance of weanling piglets, antibiotics are often used by swine producers to ensure the smooth growth of piglets (Thacker, 2013). As suggested that antibiotic associated with increased risk of sudden death in patients taking ACE inhibitors (Antoniou et al., 2010), antibiotics abuse must be reduced to avoid drug resistance and environmental impact. Thus, alternative strategies of in‐feed additives must be developed for pig production (Bi et al., 2017).

In Taiwan, natural products are widely used, considering their low toxicity and easy availability. The aim of this study was to screen natural products targeting the ACE‐2 receptor that could be an alternative antibiotic strategy for treating respiratory allergies and pneumonia in weanling piglets.

## 2 MATERIALS AND METHODS

### 2.1 Feed additives preparation

The study screened feed additives targeting the ACE‐2 receptor that could be an alternative antibiotic strategy for treating pneumonia and diarrhea in weanling piglets. We proposed a natural product "Guizhi Li‐Zhong Tang Extract Granules (GLZ)" as an alternative antibiotic feed additive for weanling piglets. GLZ is a herbal formula, which was obtained from Sun‐Ten Pharmaceutical Company (New Taipei City, Taiwan). The bioactive marker substances of GLZ were analyzed using 3D chromatographic fingerprint analysis through high‐performance liquid chromatography (HPLC, Thermo Fisher Scientific Inc.); the substances were graded with acetonitrile and methanol (Burdick & Jackson Korea, Seoul, Korea) and were qualitatively determined within 70 min under the selected HPLC condition. The ingredients of GLZ were dissolved in purified water provided by the Milli‐Q water purification system (Millipore).

### 2.2 Acute toxicity of GLZ

The maximum tolerated single dose of GLZ was determined in both sexes of rats to be 37.50 g/kg, which is equivalent to 63 times human clinical dose. The administration of GLZ at this dose demonstrated that it did not cause any signs of toxicity and mortality in the animals during 2 weeks of observation. There was no abnormal behavior observed among the animals in terms of salivation, diarrhea, hyperexcitability, respiratory distress, and mortality for the first 30 min and subsequent hours after that for 2 weeks compared to the normal control group. Therefore, it is concluded that GLZ at a lower dosage of 37.50 g/kg is safe to be administered to the animals.

### 2.3 Animal preparation

A total of 94 4‐week‐old male weanling piglets were obtained from Dafeng Ranch (Yunlin County, Taiwan). All piglets were maintained in an animal facility under specific pathogen‐free conditions at a constant temperature of 22°C ± 2°C under a 12‐hr light/dark cycle. They were given ad libitum access to water and food. To achieve completely antibiotic‐free feed, the natural product GLZ was added to the feed at 0.2%. Furthermore, a sham group and 0.2% tilmicosin antibiotic (TAB) group were maintained for comparison. The treatment was continued for 6 weeks. All animal experiments were approved by the Institutional Animal Care and Use Committee of our university (Protocol number: NTNU Animal Experiments No. 109007).

### 2.4 Blood test

After the final procedure, weanling piglets were immediately sacrificed under mild anesthesia. Blood samples were analyzed using a blood smear, which is a common practice for evaluating the health status of an animal. The blood samples were transferred to a blood sampling tube with heparin (Terumo Inc., Tokyo, Japan) and centrifuged for 15 min at 3,000 rpm at 4°C to separate the serum, which was collected and stored at −20°C until analysis. All the porcine serum samples were analyzed using a sandwich immunoglobulin A (IgA) pig enzyme‐linked immunosorbent assay (ELISA) kit (ab190536, Abcam) and a QuickDetect^TM^ IgE, immunoglobulin G (IgG) ELISA kit (E4472‐100 and E4476‐100; BioVision). All ELISA methods were conducted according to the manufacturer's instructions. After adding the stop solution, absorbance optical density (OD) was read at 450 nm by using a Multiskan™ GO Microplate Spectrophotometer reader (Thermo Scientific™). The OD value of the blank control was set as zero.

### 2.5 Immunohistochemistry staining

After perfusion of the heart with phosphate buffer, the respiratory tissues (trachea, bronchus, and lung) of weaned piglets were removed and then fixed in 4% formaldehyde. The tissue samples of the trachea, bronchus, and lung were embedded in paraffin, and then, 5‐μm sections were obtained. These sections were mounted on a glass slide for staining. Hematoxylin and eosin (H&E) staining was performed using a kit‐based method (Sigma‐Aldrich Corporation) to evaluate the respiratory tissue morphology. Moreover, immunohistochemistry (IHC) was performed using ACE‐2 antibody (Cell Signaling Technology Inc.) to evaluate ACE‐2 expression of the respiratory tissues. To observe ACE‐2 expression in the IHC‐stained respiratory tissues of weaned piglets, 3,3'‐diaminobenzidine chromogen (Novolink^TM^ polymer detection system l) followed by counterstaining with hematoxylin (Novolink^TM^ polymer detection system l) was performed.

### 2.6 Western blotting

Proteins of the respiratory tissues (trachea, bronchus, and lung) of weaned piglets were extracted using a buffer containing 50 mM Tris‐HCl (Bionovas Inc.), 150 mM NaCl (Sigma‐Aldrich Corporation), 1 mM ethylenediaminetetraacetic acid (Sigma‐Aldrich Corporation), 1 mM ethylene glycol tetraacetic acid (Bionovas Inc.), 0.1% sodium dodecyl sulfate (Bionovas Inc.), 0.5% sodium deoxycholate (Bionovas Inc.), 1% Triton X‐100, and a protease inhibitor cocktail (Sigma‐Aldrich Corporation). Then, the extracted protein was separated using 10% sodium lauryl sulfate–polyacrylamide gel electrophoresis (Bionovas Inc.) and blotted onto a nitrocellulose membrane. The membrane was treated with antibodies against ACE‐2 antibodies (Cell Signaling Technology Inc.) and β‐actin (Thermo Fisher Scientific Inc.). Antirabbit IgG‐horseradish peroxidase (HRP), antimouse IgG‐HRP (1:5,000 dilution, PerkinElmer Inc.), and antigoat IgG‐HRP (1:5,000 dilution, Enzo Life Sciences Inc., using Farmingdale, USA) were used as secondary antibodies. Chemiluminescent substrates (GE Inc. Healthcare Life Sciences) were used to detect immune complexes. LAS‐4000 (GE Inc. Healthcare Life Sciences) was used to detect chemiluminescence. ImageJ software (version 1.48t, Wayne Rasband) was used to evaluate the optical density of the Western blot.

### 2.7 Statistical analysis

All data are expressed as mean ± standard error. Statistical analysis was conducted using one‐way ANOVA followed by the Student–Newman–Keuls multiple comparison posttest. *p* < .05 was considered significant.

## 3 RESULTS

### 3.1 Chromatographic fingerprint analysis of GLZ

**FIGURE 1:**
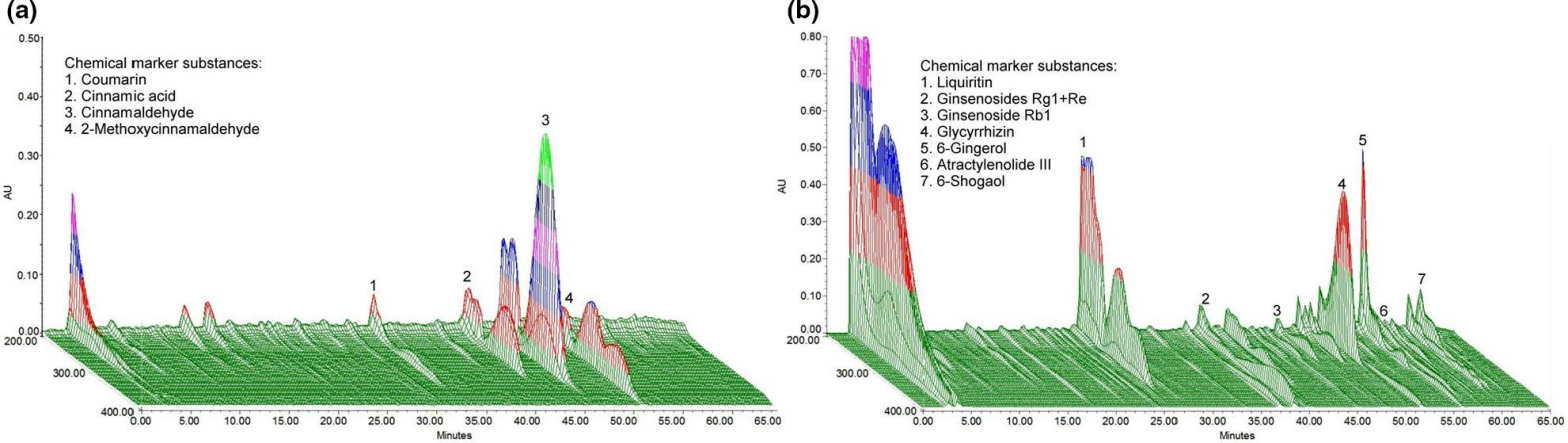
Chromatographic fingerprints of the dietary herbal formula of GLZ by using HPLC. Bioactive marker substances were as follows for (a) Guizhi: (1) coumarin, (2) cinnamic acid, (3) cinnamaldehyde, and (4) 2‐methoxycinnamaldehyde; and (b) Li‐Zhong Tang: (1) liquiritin, (2) ginsenosides Rg1 + Re, (3) ginsenoside Rb1, (4) glycyrrhizin, (5) 6‐gingerol, (6) atractylenolide III, and (7) 6‐shogaol. Bioactive marker substances of GLZ ingredients were qualitatively determined within 70 min under the selected Liquid chromatography–mass spectrometry (LC/MS) condition. AU, arbitrary perfusion units

The chromatographic fingerprint analysis results of GLZ are shown in Figure 1. Guizhi and Li‐Zhong Tang are the two main herbal ingredients of GLZ, whereas Guizhi, atractylodes, ginseng, ginger, and licorice are the other herbal ingredients of GLZ. As suggested in Figure 1, the bioactive marker substances for Guizhi are coumarin, cinnamic acid, cinnamaldehyde, and 2‐methoxycinnamaldehyde (Figure 1a), whereas those for Li‐Zhong Tang are liquiritin, ginsenosides Rg1 + Re, ginsenoside Rb1, glycyrrhizin, 6‐gingerol, atractylenolide III, and 6‐shogaol (Figure 1b).

### 3.2 Comparison of respiratory system morphology of piglets with sham, TAB, and GLZ treatments

**FIGURE 2:**
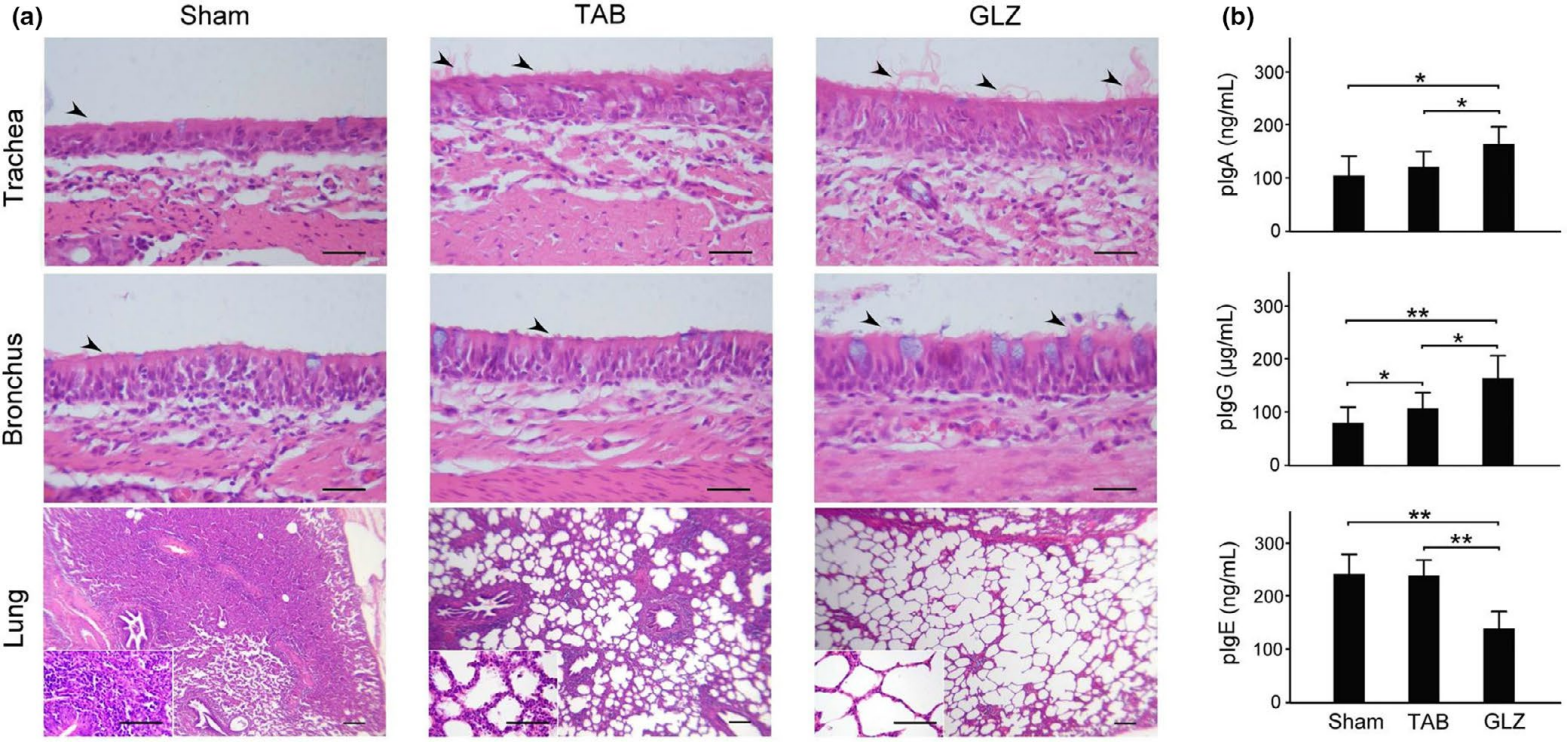
GLZ treatment alleviates epithelial cilia shedding and pneumonia and increases immunity in weanling piglets. (a) Representative H&E stains of trachea, bronchus, and lung of weanling piglets with sham, TAB, and GLZ treatments at the 10th week of age. Epithelial cilia were fragmentary in the tracheal and bronchial tissues of the sham group but were complete in the TAB and GLZ groups (marked with arrows); scale bar = 30 μm. In the lung tissue, pneumonia was severe in the sham and TAB groups but was not observed in the GLZ group; scale bar = 150 μm. Enlarged views of the lung tissue are shown in the lower left corner; scale bars = 30 μm. (b) Comparisons of IgA, IgG, and IgE in the blood of weanling piglets with sham, TAB, and GLZ treatments at the 10th week of age. Weanling piglets with GLZ treatment showed significantly higher IgA and IgG but significantly lower IgE than those with sham and TAB treatments. All data are shown as mean ± standard error of mean (*SEM*) (**p < .01, *p < .05, two‐way ANOVA followed by Student–Newman–Keuls multiple comparison posttest)

Morphology of the tracheal, bronchial, and lung tissue among weanling piglets with sham, TAB, and GLZ treatments was examined through H&E staining (Figure 2a). Weanling piglets with sham treatment showed fragmentary morphology in the epithelial cilia of the tracheal and bronchial tissues, whereas those with TAB and GLZ treatments showed complete epithelial cilia in their tracheal and bronchial tissues (Figure 2a, trachea and bronchia). Furthermore, weanling piglets with sham treatment showed severe pneumonia in their lung tissue, whereas those with TAB treatment showed partial pneumonia, and those with GLZ treatment showed normal alveolar morphology (Figure 2a, lung). Our results revealed GLZ as an alternative feed additive with potential protective effects on the respiratory system for weanling piglets.

### 3.3 Comparison of immunity among piglets with sham, TAB, and GLZ treatments

Immunities of IgA, IgG, and IgE in the blood among weanling piglets with sham, TAB, and GLZ treatments at the 10th week of age are evaluated in Figure 2b by using IgA pig enzyme‐linked immunosorbent assay (ELISA) kit (ab190536, Abcam) and a QuickDetect^TM^ IgE, IgG ELISA kit (E4472‐100 & E4476‐100; BioVision). Those weanling piglets with GLZ treatment significantly increased IgA and IgG (Figure 2b, *p* < .01–0.05), but significantly decreased IgE (Figure 2b, *p* < .01–0.05) than those of weanling piglets with sham and TAB treatments. Our results revealed that alternative CHM of GLZ treatment should enhance immunity function and relieve respiratory allergies for weanling piglets.

### 3.4 Comparison of ACE‐2 expression in the respiratory systems of piglets with sham, TAB, and GLZ treatments

**FIGURE 3:**
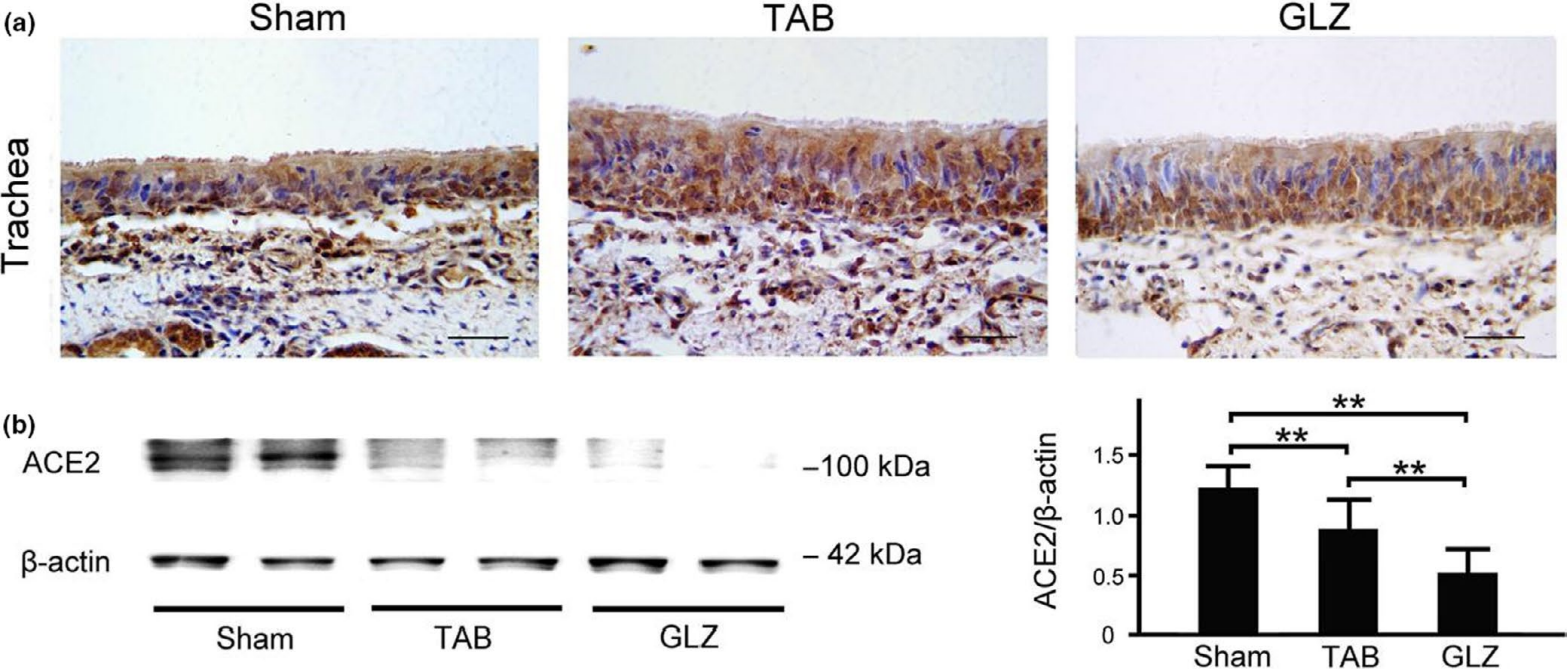
GLZ treatment reduces ACE‐2 expression in the tracheal tissue of weanling piglets. (a) Representative IHC stains of ACE‐2 expression in the tracheal tissue of weanling piglets with sham, TAB, and GLZ treatments at the 10th week of age. In the tracheal tissue, IHC expression of ACE‐2 (indicated with dark brown color) in weanling piglets with GLZ treatment was lower than that in those with sham and TAB treatments; scale bar = 30 μm. (b) Using Western blotting, ACE‐2 expression in the tracheal tissue of weanling piglets with sham, TAB, and GLZ treatments at the 10th week of age. In the tracheal tissue, quantified expression of ACE‐2 in weanling piglets with GLZ and TAB treatments was significantly lower than that in those with sham treatment. All data are shown as mean ± *SEM* (**p < .01, one‐way ANOVA followed by Student–Newman–Keuls multiple comparison posttest). Experiments were repeated thrice for each treatment

**FIGURE 4:**
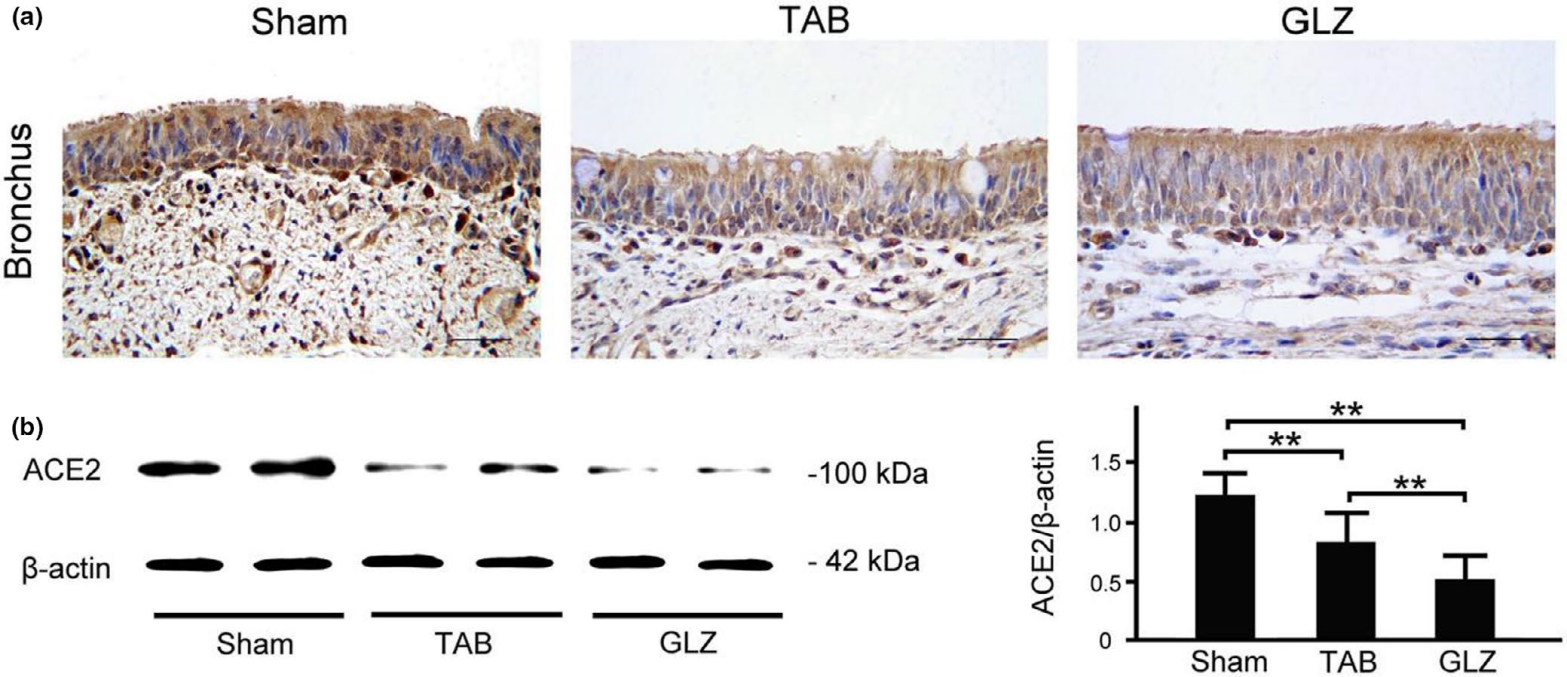
GLZ treatment reduces ACE‐2 expression in the bronchial tissue of weanling piglets. (a) Representative IHC stains of ACE‐2 expression in the bronchial tissue of weanling piglets with sham, TAB, and GLZ treatments at the 10th week of age. In the bronchial tissue, IHC expression of ACE‐2 (indicated with dark brown color) in weanling piglets with GLZ treatment was lower than that in those with sham and TAB treatments; scale bar = 30 μm. (b) Using Western blotting, ACE‐2 expression in the bronchial tissue of weanling piglets with sham, TAB, and GLZ treatments at the 10th week of age. In the bronchial tissue, quantified expression of ACE‐2 in weanling piglets with GLZ treatment was significantly lower than that in those with sham and TAB treatments. All data are shown as mean ± *SEM* (**p < .01, one‐way ANOVA followed by Student–Newman–Keuls multiple comparison posttest). Experiments were repeated thrice for each treatment

ACE‐2 expression in the tracheal, bronchial, and lung tissues was evaluated among weanling piglets with sham, TAB, and GLZ treatments at the 10th week of age by using IHC staining and Western blotting (Figures 3 and 4). In the tracheal tissue, weanling piglets with GLZ treatment showed lower ACE‐2 expression than those with sham and TAB treatments (Figure 3a). Furthermore, the quantified expression of ACE‐2 in the tracheal tissue of weanling piglets with GLZ treatment was significantly lower than the expression in those with sham and TAB treatments (Figure 3b, *p* < .01–.05). Similarly, in the bronchial tissue, weanling piglets with GLZ treatment showed lower ACE‐2 expression than those with sham and TAB treatments (Figure 4a), and the quantified expression of ACE‐2 in the bronchial tissue of weanling piglets with GLZ treatment was significantly lower than the expression in those with sham and TAB treatments (Figure 4b, *p* < .01–.05). The results revealed that alternative feed additives of GLZ had protective effects on the respiratory tract by reducing ACE‐2 expression and preventing viral infection in weanling piglets.

**FIGURE 5:**
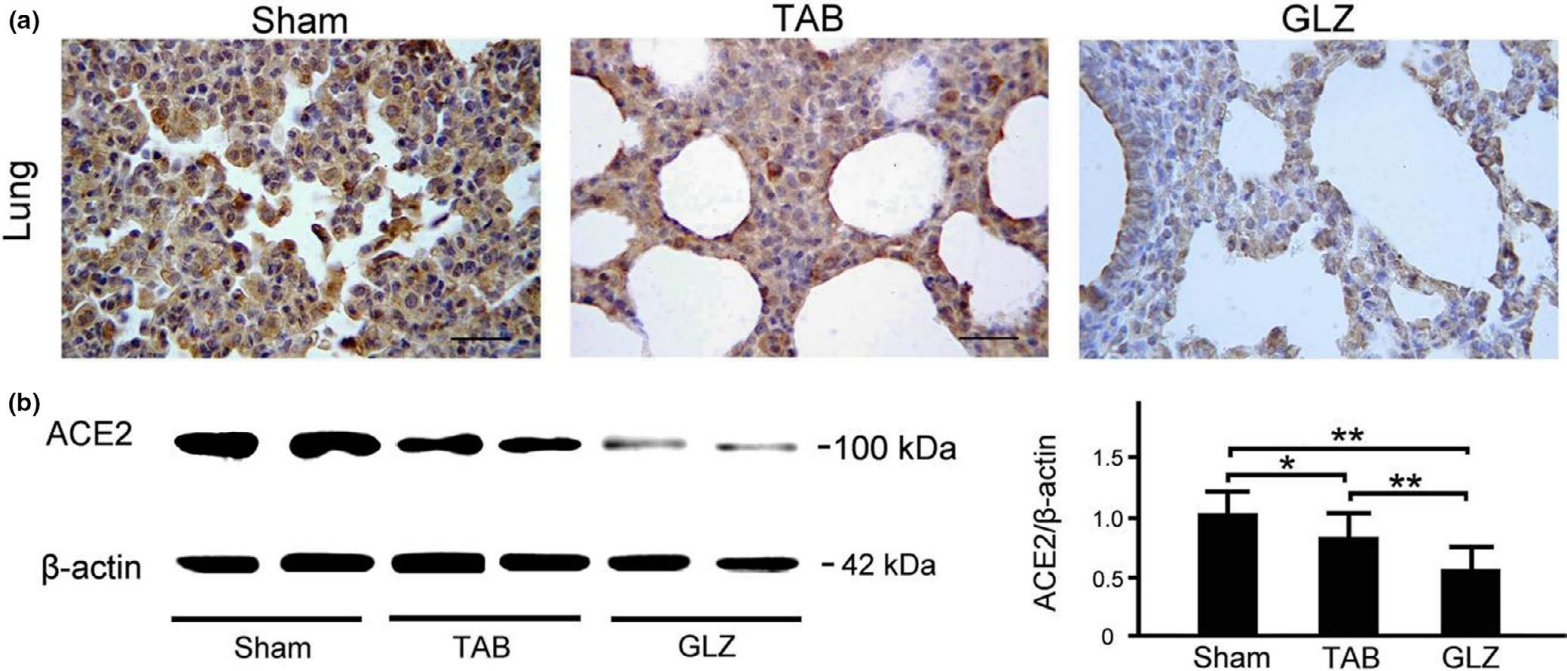
GLZ treatment reduces ACE‐2 expression in the lung tissue of weanling piglets. (a) Representative IHC stains of ACE‐2 expression in the lung tissue of weanling piglets with sham, TAB, and GLZ treatments at the 10th week of age. In the lung tissue, IHC expression of ACE‐2 (indicated with dark brown color) in weanling piglets with GLZ treatment was lower than that in those with sham and TAB treatments; scale bar = 30 μm. (b) Using Western blotting, ACE‐2 expression in the lung tissue of weanling piglets with sham, TAB, and GLZ treatments at the 10th week of age. In the lung tissue, quantified expression of ACE‐2 in weanling piglets with GLZ treatment was significantly lower than that in those with sham and TAB treatments. All data are shown as mean ± *SEM* (**p < .01, *p < .05, one‐way ANOVA followed by Student–Newman–Keuls multiple comparison posttest). Experiments were repeated thrice for each treatment

In the lung tissue, weanling piglets with sham and GLZ treatments showed lower ACE‐2 expression than those with TAB treatment (Figure 5a), and the quantified expression of ACE‐2 in the lung tissue of weanling piglets with GLZ was significantly lower than the expression in those with sham and TAB treatment treatments (Figure 5b, *p* < .01–.05). The results revealed that the alternative feed additive of GLZ had protective effects on the lung by decreasing ACE‐2 expression and alleviating pneumonia in weanling piglets.

### 3.5 Comparison of pneumonia incidence of weanling piglets with sham, TAB, and GLZ treatments

**FIGURE 6:**
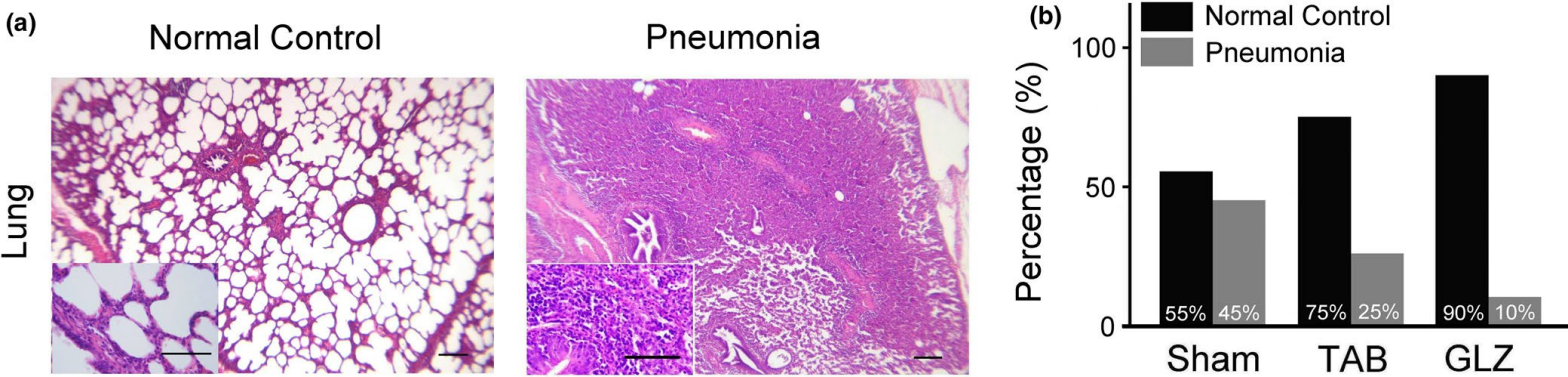
GLZ treatment reduces pneumonia incidence in the lung tissue of weanling piglets. (a) Representative H&E stains of lung of weanling piglets with normal and pneumonia; scale bar = 150 μm. Enlarged views of the lung tissue are shown in the lower left corner; scale bars = 30 μm. (b) Comparisons of the percentage of weaning piglets suffer from the pneumonia among sham, TAB, and GLZ treatments at the 10th week of age

Figure 6 evaluates pneumonia incidence among weanling piglets with sham, TAB, and GLZ treatments at the 10th week of age by using IHC staining (Figure 6a). The results revealed that alternative feed additives of GLZ had protective effects on the on the lung tissue by reducing and preventing pneumonia in weanling piglets. About 45% of weanling piglets with sham treatment showed severe pneumonia in their lung tissue, whereas 25% of weanling piglets those with TAB treatment and only 10% of weanling piglets with GLZ treatment showed partial pneumonia in this study (Figure 6b). Our results revealed GLZ as an alternative feed additive with potential protective effects on the respiratory system for weanling piglets.

## 4 DISCUSSION

In Taiwan, some weanling piglets develop pneumonia with a high expression of ACE‐2 in their respiratory system. Without antibiotic or alternative feed additive treatments, these weanling piglets showed fragmentary morphology in the epithelial cilia of the tracheal and bronchial tissues and severe pneumonia in their lung tissue. However, weanling piglets with antibiotic or alternative feed additive treatments showed complete epithelial cilia in their tracheal and bronchial tissues and normal alveolar morphology in their lung tissue (Figure 2).

ACE is crucial for regulating the renin–angiotensin system, which can cleave angiotensin‐I to produce angiotensin‐II and then inactivate the vasodilator peptide bradykinin (Corvol et al., 1995). ACE inhibition alters blood pressure (Schiffrin, 2002). Studies have reported that certain flavanol‐rich foods can reduce blood pressure and may act as pharmacological ACE inhibitors (Actis‐Goretta et al., 2003). In this study, GLZ was selected as an alternative feed additive for piglets. GLZ is composed of five medicines, namely Guizhi, ginseng, dried ginger, *Atractylodes macrocephala*, and roasted licorice. According to *Treatise on Febrile Diseases*, GLZ is used to treat people with spleen function deficiency and continuous diarrhea caused by cold pathogens. Studies have reported that consuming ginseng alone may increase blood pressure. However, after consuming GLZ, patients may have lower blood pressure. Thus, GLZ may act as a pharmacological ACE inhibitor. In addition, ACE‐2 may play an important role in acute lung injury induced by influenza viruses, such as H1N1 and H5N1 (Liu et al., 2014; Zou et al., 2014). Cinnamaldehyde, the active ingredient of GLZ, has been confirmed to inhibit H1N1 infection in animals and SARS‐CoV‐2 Infection (Hayashi et al., 2007; Silva et al., 2020).

ACE‐2 is the main host cell receptor of 2019‐nCoV that plays a crucial role in the entry of virus into the cell. Our results showed that ACE‐2 could be expressed in the tracheal, bronchial, and lung tissues and was highly enriched in epithelial cells in weanling piglets. Moreover, increased ACE‐2 expression in tracheal and bronchial epithelial cells might be a high‐risk route of virus infection. We selected GLZ as a feed additive for piglets. Our results revealed that piglets with GLZ treatment showed a significantly lower expression of ACE‐2 in the tracheal, bronchial, and lung tissues than those with sham and TAB treatments. As described earlier, Guizhi is one of the important components of GLZ. Guizhi has antibacterial effects on the influenza virus, pneumococcus, dysentery bacillus, *Escherichia coli*, and others and is extensively used in the clinical treatment of influenza (Fu et al., 2018). After viral infection, Guizhi showed better antiviral effect under normal conditions. Piglets with GLZ treatment showed a significantly lower expression of ACE‐2 in the tracheal, bronchial, and lung tissues than those with sham and TAB treatments (Figures 3, 4, 5). In addition, piglets with GLZ treatment showed protective effects on the lung tissue by reducing and preventing pneumonia in weanling piglets (Figure 6). Thus, we suggested that GLZ may have an antiviral effect through decreased ACE‐2 expression in the respiratory system of weanling piglets.

IgA plays a crucial role in the immune function of mucous membranes. IgG is the main potentiator of humoral immunity in the host extracellular fluids, including blood, lymph, and saliva. IgE plays a crucial role for patients with allergy (Amarasekera, 2011). Our results showed that piglets with GLZ treatment showed a significantly higher expression of IgA and IgG but a lower expression of IgE than those with sham and TAB treatments (Figure 2). This immunological evidence supports that piglets with GLZ treatment develop immunity for preventing viral or bacterial infection and reducing allergies in their respiratory system.

Weanling piglets possess limited natural resistance against many infectious diseases; hence, the piglet industry relies on antibiotics or related feed additives to increase the disease resistance of piglet flocks. Antibiotic use in piglet feed improves performance and decreases morbidity in swine. However, consumer pressure related to the potential development of antibiotic‐resistant bacteria in the human population has resulted in the development of antibiotic‐alternative feed additives in animal rations, which may also improve swine performance (Buchanan et al., 2008; Phillips et al., 2004). The antiviral properties of GLZ, exerted by decreasing ACE‐2 expression in the respiratory system, may offer additional, important mechanisms by which piglets may be protected against many prevalent respiratory diseases. Thus, feed additives such as GLZ seem to be good alternatives to antibiotic medications because of their biological properties.

## 5 CONCLUSION

**FIGURE 7:**
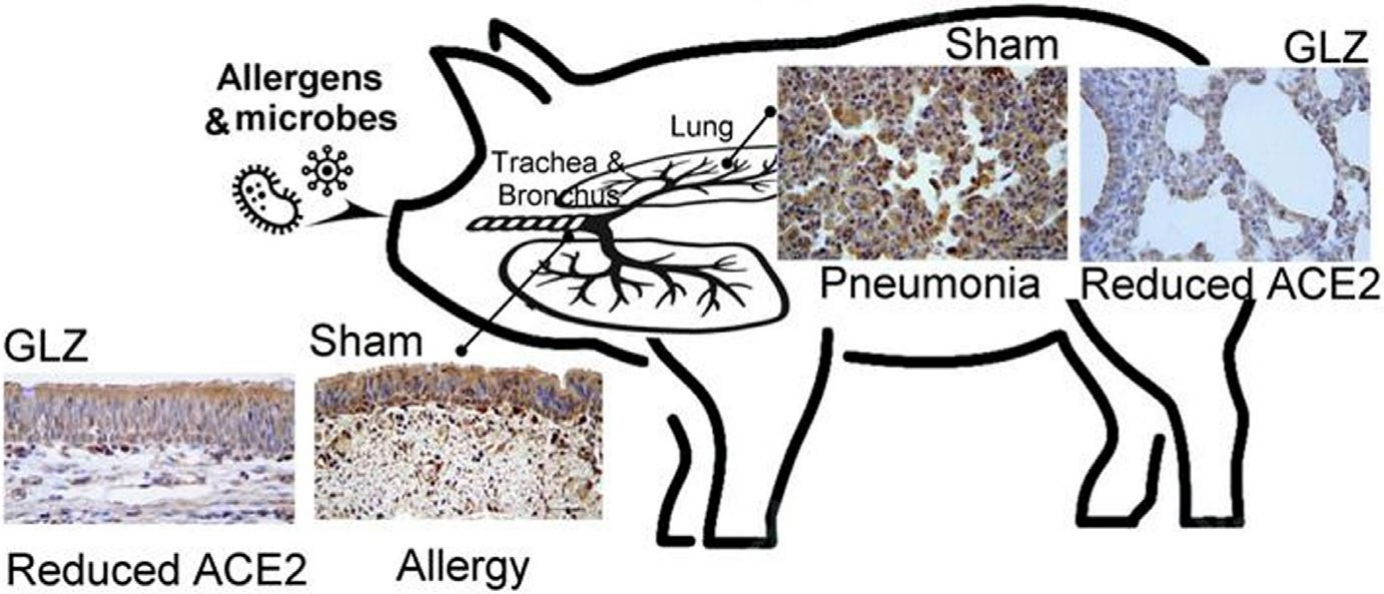
Schematic diagram illustrates that dietary herbal GLZ treatment can defend the respiratory system of weanling piglets from allergies and respiratory symptoms because of reduced ACE‐2 expression in the trachea, bronchus, and lung tissue

In weanling piglets, feeding antibiotics did not have additional benefits over feed additives. However, feeding feed additives of GLZ tended to have an additive alleviative effect over TAB treatment alone on respiratory allergies and pneumonia in the respiratory systems of weanling piglets. As suggested from our study, feeding GLZ clearly demonstrated beneficial effects in alleviating respiratory allergies and pneumonia in the respiratory systems of weanling piglets through the inhibition of ACE‐2 expression compared with sham and antibiotic treatments (Figure 7). Additionally, GLZ treatment caused significantly higher IgA and IgG but lower IgE levels than sham and TAB treatments. Our results support that GLZ is an alternative antibiotic feed additive for enhancing immune protection by increasing IgA and IgG levels, alleviating respiratory allergies and pneumonia by decreasing ACE‐2 and IgE expressions, and preventing viral infection in weanling piglets. The present results indicate that GLZ is a potential antibiotic alternative in piglet diets.

## CONFLICT OF INTEREST

The authors have no conflicts of interest to declare.

## ETHICAL APPROVAL

All animal experiments were approved by the Institutional Animal Care and Use Committee of our university (protocol number: NTNU Animal Experiments no. 109007).

## Supporting information

Tables S1 and S2

## Data Availability

Research data are not shared.
