# Supplementary material for: Alternative antibiotic feed additives alleviate pneumonia with inhibiting ACE‐2 expression in the respiratory system of piglets
Source: Food Sci Nutr. 2020 Dec 27;9(2):1112–20. doi: 10.1002/fsn3.2089 (PMC7866577; doi:10.1002/fsn3.2089)
Supplement: Supplementary file 1 — Tables S1 and S2 [file FSN3-9-1112-s001.pdf]

## Supplementary table 1:

The content of bioactive marker substances in GLZ

| Composition | content (mg/g) |
|---|---|
| Atractylodes macrocephala | 889 |
| Panax ginseng | 889 |
| Glycyrrhiza uralensis | 889 |
| Zingiber officinale | 667 |
| Cinnamomum cassia Presl | 402 |

## Supplementary table 2:

Effect of GLZ on biochemical parameters in porcine blood

| Biochemical parameters | Sham | TAB | GLZ |
|---|---|---|---|
| Glucose (mg/dL) | 134 ± 36 | 105± 26 | 118 ± 3.0 |
| Creatinine (mg/dL) | 0.8 ± 0.2 | 0.8 ± 0.1 | 0.6 ± 0.1 |
| Blood urea nitrogen (mg/dL) | 3.0 ± 1.0 | 5.0± 1.0* | 3.0 ± 1.0 |
| Total protein (g/dL) | 7.2 ± 0.7 | 6.5 ± 0.3 | 7.8 ± 1.0 |
| Globulin (g/dL) | 4.4 ± 0.4 | 3.8 ± 0.5 | 4.3 ± 0.5 |
| Alanine transamidation (U/L) | 181 ± 128 | 75.0 ± 10 | 91.0 ± 28 |
| Alkaline phosphatase (U/L) | 96 ± 30 | 213± 121* | 29 ± 24 |

*: $p<0.05$
